# Validation List no. 221: valid publication of new names and new combinations effectively published outside the IJSEM

**DOI:** 10.1099/ijsem.0.006562

**Published:** 2025-01-31

**Authors:** Aharon Oren, Markus Göker

**Affiliations:** 1 The Institute of Life Sciences, The Hebrew University of Jerusalem, The Edmond J. Safra Campus, 9190401 Jerusalem, Israel; 2 Leibniz Institute DSMZ – German Collection of Microorganisms and Cell Cultures, Inhoffenstrasse 7B, 38124 Braunschweig, Germany

The purpose of this list is to publish in the *International Journal of Systematic and Evolutionary Microbiology* (*IJSEM*) those new names and new combinations that were effectively published outside the *IJSEM* as set forth in Rule 27 of the International Code of Nomenclature of Prokaryotes (ICNP) (2022 Revision) [1]. The authors and other individuals wishing to have new names and/or combinations included in future lists should send an electronic copy of the published paper to the *IJSEM* Editorial Office for confirmation that all of the other requirements for valid publication have been met. It is also a requirement of *IJSEM* and the ICNP that the authors of new species, new subspecies and new combinations provide evidence that types are deposited in two publicly accessible culture collections in two different countries. It should be noted that the date of valid publication of these new names and combinations is the date of publication of this list, not the date of the original publication of the names and combinations. The authors of the new names and combinations are given below.

The inclusion of a name on these lists validates the publication of the name and thereby makes it available in the nomenclature of prokaryotes. The inclusion of a name on these lists is part of the requirements of valid publication of an effectively published name. Indeed, some of these names may, in time, be considered later synonyms, or it may be proposed to transfer the organisms to another genus, thus necessitating the creation of a new combination. Conversely, it may be proposed to transfer an organism back to an earlier genus and to regard the basonym or an earlier new combination as the correct name instead of a more recent new combination. The ICNP does not decide on such taxonomic opinions.

**Table:** 

Name/authors	Proposed as	Nomenclatural type^1^	Priority^2^	References
*Actinomycetospora termitidis* Supong *et al*. 2024, 302	sp. nov.	Odt1-22 (=NBRC 115965=TBRC 16192)	16	[2]
*Actinoplanes kirromycinicus* Zeng *et al*. 2024, 662	sp. nov.	TPMA0078 (=NBRC 116422=TBRC 18262)	57	[3]
*Aerobium* Li *et al*. 2024, 12^3^	gen. nov.	*Aerobium aerolatum*	7	[4]
*Aerobium aerolatum* (Kämpfer *et al*. 2009) Li *et al*. 2024, 12^3^	comb. nov. [basonym: *Aquamicrobium aerolatum* Kämpfer *et al*. 2009]	Sa14 (=CCUG 57044=DSM 21857)	7	[4]
*Aliirhizobium terrae* Lin *et al*. 2024, 9^3^	sp. nov.	CC-CFT758 (=BCRC 81364=JCM 35482)	29	[5]
*Allomesorhizobium* Li *et al*. 2024, 10^3^	gen. nov.	*Allomesorhizobium alhagi*	7	[4]
*Allomesorhizobium alhagi* (Chen *et al*. 2010) Li *et al*. 2024, 10^3^	comb. nov. [basonym: *Mesorhizobium alhagi* Chen *et al*. 2010]	CCNWXJ12-2 (=ACCC 14561=HAMBI 3019)^4^	7	[4]
*Allomesorhizobium camelthorni* (Chen *et al*. 2011) Li *et al*. 2024, 11^3^	comb. nov. [basonym: *Mesorhizobium camelthorni* Chen *et al*. 2011]	CCNWXJ 40-4 (=DSM 100023=HAMBI 3020)^5^	7	[4]
*Anoxybacterium* Cao *et al*. 2024, 10^3^	gen. nov.	*Anoxybacterium hadale*	18	[6]
*Anoxybacterium hadale* Cao *et al*. 2024, 10^3,6^	sp. nov.	MT110 (=KCTC 15922=MCCC 1K04061)	18	[6]
*Aquibaculum sediminis* Xu *et al*. 2025, 9^3^	sp. nov.	EGI_FJ10229 (=GDMCC 1.4598=KCTC 8570)	61	[7]
*Archangium lansingense* corrig. Ahearne *et al*. 2023, 11^3,7^	sp. nov.	MIWBW (=ATCC TSD-326=NCCB 100916)^8^	43	[8]
*Atlantibacter* Hata *et al*. 2016, 308	gen. nov.	*Atlantibacter hermannii*	44	[9]
*Atlantibacter hermannii* (Brenner *et al*. 1983) Hata *et al*. 2016, 309^9^	comb. nov. [basonym: *Escherichia hermannii* Brenner *et al*. 1983]	CIP 103176=DSM 4560=JCM 1473=LMG 7867^10^	44	[9]
*Atlantibacter subterraneus* corrig. (Shelobolina *et al*. 2005) Hata *et al*. 2016, 309^11^	comb. nov. [basonym: *Salmonella subterranea* Shelobolina *et al*. 2005]	FRCl (=ATCC BAA-836=CIP 109002=DSM 16208)	44	[9]
*Bacillus yapensis* Xu *et al*. 2020, 394	sp. nov.	XXST-01 (=JCM 33181=MCCC 1A14143)	4	[10]
*Bacteroides vicugnae* Miller *et al*. 2024, 5^3^	sp. nov.	A2-P53 (=CCM 9377=NRRL B 65693)^12^	58	[11]
*Bartonella apihabitans* Liu *et al*. 2022, 7^3^	sp. nov.	W8097 (=ACCC 62056=JCM 35029)	28	[12]
*Bartonella choladocola* Liu *et al*. 2022, 7^3^	sp. nov.	W8125 (=ACCC 62057=JCM 35030)	28	[12]
*Borborobacter* Li *et al*. 2024, 11^3^	gen. nov.	*Borborobacter arsenicus*	7	[4]
*Borborobacter arsenicus* (Mu *et al*. 2019) Li *et al*. 2024, 11^3^	comb. nov. [basonym: *Pseudaminobacter arsenicus* Mu *et al*. 2019]	CB3 (=CCTCC AB2016116=KCTC 52625)	7	[4]
*Bovifimicola* Hitch *et al*. 2021, 9^3^	gen. nov.	*Bovifimicola ammoniilytica*	40	[13]
*Bovifimicola ammoniilytica* Hitch *et al*. 2021, 10^3^	sp. nov.	Sanger_97 (=DSM 102166=JCM 37190)^13^	40	[13]
*Brotomerdimonas* Hitch *et al*. 2021, 10^3^	gen. nov.	*Brotomerdimonas butyrica*	40	[13]
*Brotomerdimonas butyrica* Hitch *et al*. 2021, 10^3^	sp. nov.	Sanger_12 (=DSM 102176=LMG 29485)	40	[13]
*Chengkuizengella axinellae* Moon *et al*. 2024, 8^3,14^	sp. nov.	2205SS18-9 (=KACC 23238=LMG 33063)	45	[14]
*Clostridium mucosae* Afrizal *et al*. 2022, e15^3^	sp. nov.	PG-426-IM-1 (=DSM 100503=JCM 35244)	26	[15]
*Coprococcus aceti* Hitch *et al*. 2021, 11^3^	sp. nov.	H2_11 (=DSM 107541=JCM 31265)	40	[13]
*Crenobacter oryzisoli* Zhang *et al*. 2024, 8^3^	sp. nov.	SG2303 (=GDMCC 1.3970=JCM 36468)	35	[16]
*Demequina capsici* Humaira *et al*. 2024, 7^3^	sp. nov.	PMTSA13 (=GDMCC 1.4451=KCTC 59028)	15	[17]
*Desertibaculum* Feng *et al*. 2024, 7^3^	gen. nov.	*Desertibaculum subflavum*	2	[18]
*Desertibaculum subflavum* Feng *et al*. 2024, 9^3^	sp. nov.	SYSU D60015 (=CGMCC 1.16256=NBRC 112952)	2	[18]
*Desulfofundulus salinus* Nazina *et al*. 2024, 17^3^	sp. nov.	435 (=DSM 23196=VKM B-1492)	11	[19]
*Enterobacter chinensis* He *et al*. 2024, 7^3^	sp. nov.	170198 (=GDMCC 1.3549=JCM 35826)	12	[20]
*Enterobacter rongchengensis* He *et al*. 2024, 8^3^	sp. nov.	170250 (=GDMCC 1.3670=JCM 36189)	12	[20]
*Faecalibacterium wellingii* Plomp and Harmsen 2024, 5^3,15^	sp. nov.	HTF-F (=DSM 117771=NCIMB 15531)	23	[21]
*Falsiroseomonas oleicola* (Wu *et al*. 2021) Liu and Xin 2024, 8^3^	comb. nov. [basonym: *Roseomonas oleicola* Wu *et al*. 2021]	ROY-5-3 (=CGMCC 1.13459=KCTC 82484)	32	[22]
*Falsiroseomonas ponticola* (Yin *et al*. 2021) Liu and Xin 2024, 8^3^	comb. nov. [basonym: *Roseomonas ponticola* Yin *et al*. 2021]	SYSU M41301 (=CGMCC 1.18613=KCTC 72726)	32	[22]
*Gallaecimonas kandeliae* Long *et al*. 2023, 901	sp. nov.	Q10 (=KCTC 92860=MCCC 1K08421)	25	[23]
*Halapricum hydrolyticum* Sorokin *et al*. 2024, 2^3^	sp. nov.	HArc-curdl5-1 (=DSM 114193=UQM 41587)	42	[24]
*Halomonas flagellata* Huang *et al*. 2023, 7^3^	sp. nov.	EGI 63088 (=CGMCC 1.19133=KCTC 92047)	3	[25]
*Halorubrum miltondacostae* Albuquerque *et al*. 2024, 8^3^	sp. nov.	RMP-11 (=CECT 30760=DSM 115521)	46	[26]
*Kumtagia* Li *et al*. 2024, 11^3^	gen. nov.	*Kumtagia ephedrae*	7	[4]
*Kumtagia ephedrae* (Liu *et al*. 2018) Li *et al*. 2024, 11^3^	comb. nov. [basonym: *Mesorhizobium ephedrae* Liu *et al*. 2018]	6GN-30 (=ACCC 60073=KCTC 62410)^16^	7	[4]
*Lacticaseibacillus jixiensis* Song *et al*. 2024, 5^3^	sp. nov.	N163-3-2 (=CCTCC AB 2024125=JCM 36999=LMG 33661)	47	[27]
*Lapidilactobacillus salsurivasis* Wang *et al*. 2024, 6^3^	sp. nov.	475-2 (=CCTCC AB 2023258=JCM 36613=LMG 33412)	34	[28]
*Lelliottia wanjuensis* Lee *et al*. 2024, 8^3,17^	sp. nov.	V104_15 (=DSM 115585=LMG 32996)	51	[29]
*Manganibacter* Li *et al*. 2024, 10^3,18^	gen. nov.	*Manganibacter manganicus*	7	[4]
*Manganibacter manganicus* (Li *et al*. 2017) Li *et al*. 2024, 10^3^	comb. nov. [basonym: *Pseudaminobacter manganicus* Li *et al*. 2017]	JH-7 (=CCTCC AB 2016107=KCTC 52258)	7	[4]
*Marinobacterium marinum* Liu *et al*. 2024, 16^3^	sp. nov.	3-1745 (=KCTC 72925=MCCC 1H00422)^19^	30	[30]
*Marinobacterium weihaiense* Liu *et al*. 2024, 15^3^	sp. nov.	A346 (=KCTC 92007=MCCC 1H00492)^20^	30	[30]
*Marivirga arenosa* Muhammad *et al*. 2024, 13^3^	sp. nov.	BKB1-2 (=InaCC B1618=KCTC 82989)	14	[31]
*Marivirga salinarum* corrig. Muhammad *et al*. 2024, 14^3,21^	sp. nov.	BDSF4-3 (=InaCC B1619=KCTC 82973)	14	[31]
*Methanospirillum purgamenti* Pradel *et al*. 2024, 20^3^	sp. nov.	J.3.6.1-F.2.7.3 (=DSM 107957=NBRC 114537)	22	[32]
*Microbacterium betulae* Paściak *et al*. 2024, 10^3^	sp. nov.	AB (=CECT 30706=PCM 3040)	31	[33]
*Microbispora aerata* (Gerber and Lechevalier 1964) Bouras and Machado 2024, 6^3^	comb. nov. [basonym: ‘*Waksmania aerata*’ Gerber and Lechevalier 1964]	ATCC 15448=DSM 43176^22^	55	[34]
*Microbispora bryophytorum* subsp. *bryophytorum* (Li *et al*. 2015) Bouras and Machado 2024, 7^3^	comb. nov. [basonym: *Microbispora bryophytorum* Li et al. 2015]	NEAU TX2-2 (=CGMCC 4.7138=DSM 46710)	54	[34]
*Microbispora bryophytorum* subsp. *camponoti* Bouras and Machado 2024, 8^3^	subsp. nov.	2C-HV3 (=CGMCC 4.7281=DSM 100527)	54	[34]
*Micromonospora palythoicola* Kanchanasin *et al*. 2024, 9^3^	sp. nov.	S2-005 (=NBRC 116545=TBRC 18343)	53	[35]
*Micromonospora sicca* Carro *et al*. 2024, 6^3^	sp. nov.	4G51 (=LMG 30756=PCM 3031)	33	[36]
*Muricoccus aeriglobus* (Lee and Jeon 2018) Liu and Xin 2024, 8^3^	comb. nov. [basonym: *Roseomonas aeriglobus* Lee and Jeon 2018]	KER25-12 (=JCM 32049=KACC 19282)	32	[22]
*Muricoccus aerilatus* (Yoo *et al*. 2008) Liu and Xin 2024, 8^3^	comb. nov. [basonym: *Roseomonas aerilata* Yoo *et al*. 2008]	5420S-30 (=DSM 19363=KACC 12521)	32	[22]
*Muricoccus harenae* (Deng *et al*. 2020) Liu and Xin 2024, 9^3^	comb. nov. [basonym: *Roseomonas harenae* Deng *et al*. 2020]	CPCC 101081 (=KCTC 62852=NBRC 113512)	32	[22]
*Muricoccus nepalensis* (Chaudhary and Kim 2017) Liu and Xin 2024, 9^3^	comb. nov. [basonym: *Roseomonas nepalensis* Chaudhary and Kim 2017]	G-3-5 (=JCM 31470=KACC 18908)^23^	32	[22]
*Muricoccus pecuniae* (Lopes *et al*. 2011) Liu and Xin 2024, 9^3^	comb. nov. [basonym: *Roseomonas pecuniae* Lopes *et al*. 2011]	N75 (=DSM 25268=LMG 25481)^24^	32	[22]
*Muricoccus radiodurans* (Kim *et al*. 2018) Liu and Xin 2024, 9^3^	comb. nov. [basonym: *Roseomonas radiodurans* Kim *et al*. 2018]	17Sr1-1 (=KCTC 52899=NBRC 112872)	32	[22]
*Muricoccus vinaceus* (Zhang *et al*. 2008) Liu and Xin 2024, 9^3^	comb. nov. [basonym: *Roseomonas vinacea* Zhang *et al*. 2008]	CPCC 100056 (=DSM 19362=KCTC 22045)^25^	32	[22]
*Myxococcus landrumensis* corrig. Ahearne *et al*. 2023, 11^3,26^	sp. nov.	SCHIC03 (=ATCC TSD-327=NCCB 100915)^27^	43	[8]
*Nannocystis punicea* corrig. Ahearne *et al*. 2023, 12^3,28^	sp. nov.	FL3 (=ATCC TSD-332=NCCB 100918)^29^	43	[8]
*Natronoglomus* Sorokin *et al*. 2024, 12^3^	gen. nov.	*Natronoglomus mannanivorans*	37	[37]
*Natronoglomus mannanivorans* Sorokin *et al*. 2024, 12^3^	sp. nov.	AArc-m2/3/4 (=JCM 34861=UQM 41565)	37	[37]
*Natronosalvus hydrolyticus* Sorokin *et al*. 2024, 7^3^	sp. nov.	AArc-curdl1 (=JCM 34865=UQM 41566)	36	[38]
*Natronospira bacteriovora* Sorokin *et al*. 2024, 2^3^	sp. nov.	AB-CW4 (=JCM 35397=UQM 41580)	38	[38]
*Natronospira elongata* Sorokin *et al*. 2024, 2^3^	sp. nov.	AB-CW1 (=JCM 35396=UQM 41579)	38	[39]
*Natronospiraceae* Sorokin *et al*. 2024, 2^3^	fam. nov.	*Natronospira*	38	[39]
*Natronospirales* Sorokin *et al*. 2024, 2^3^	ord. nov.	*Natronospira*	38	[39]
*Neoaquamicrobium* Li *et al*. 2024, 11^3^	gen. nov.	*Neoaquamicrobium sediminum*	7	[4]
*Neoaquamicrobium microcysteis* (Jung *et al*. 2021) Li *et al*. 2024, 12^3^	comb. nov. [basonym: *Mesorhizobium microcysteis* Jung *et al*. 2021]	MaA-C15 (=JCM 33503=KACC 21226)	7	[4]
*Neoaquamicrobium sediminum* (Yuan *et al*. 2016) Li *et al*. 2024, 12^3^	comb. nov. [basonym: *Mesorhizobium sediminum* Yuan *et al*. 2016]	YIM M12096 (=CCTCC AB 2014219=KCTC 42205)	7	[4]
*Neomesorhizobium* Li *et al*. 2024, 11^3^	gen. nov.	*Neomesorhizobium albiziae*	7	[4]
*Neomesorhizobium albiziae* (Wang *et al*. 2007) Li *et al*. 2024, 12^3^	comb. nov. [basonym: *Mesorhizobium albiziae* Wang *et al*. 2007]	CCBAU 61158 (=DSM 21822=LMG 23507)^30^	7	[4]
*Neoroseomonas rubea* (Lee and Whang 2022) Liu and Xin 2024, 8^3^	comb. nov. [basonym: *Roseomonas rubea* Lee and Whang 2022]	MO17 (=KACC 19933=NBRC 114495)	32	[22]
*Novosphingobium pituita* Ikarashi *et al*. 2024, 6^3,31^	sp. nov.	IK01 (=DSM 116658=NBRC 116408)	60	[40]
*Ollibium* Li *et al*. 2024, 10^3^	gen. nov.	*Ollibium composti*	7	[4]
*Ollibium composti* (Lin *et al*. 2020) Li *et al*. 2024, 10^3,32^	comb. nov. [basonym: *Mesorhizobium composti* Lin *et al*. 2020]	CC-YTH430 (=BCRC 81024=JCM 31762)	7	[4]
*Paracraurococcus lichenis* Kingkaew *et al*. 2024, 8^3^	sp. nov.	LOR1-02 (=JCM 33121=NBRC 112776=TISTR 2503)	48	[41]
*Porcipelethomonas* Hitch *et al*. 2021, 13^3^	gen. nov.	*Porcipelethomonas ammoniilytica*	40	[13]
*Porcipelethomonas ammoniilytica* Hitch *et al*. 2021, 13^3^	sp. nov.	Sanger_90 (=DSM 102167=JCM 37191)	40	[13]
*Providencia hangzhouensis* Dong *et al*. 2024, 1^3^	sp. nov.	PR-310 (=CCTCC AB 2023265=NBRC 116613)	9	[42]
*Providencia xianensis* Dong *et al*. 2024, 1465	sp. nov.	23021821 (=CCTCC AB 2023264=NBRC 116615)	10	[43]
*Providencia zhijiangensis* Dong *et al*. 2024, 1^3^	sp. nov.	D4759 (=CCTCC AB 2023263=NBRC 116614)	8	[44]
*Pseudaminobacter soli* (Nguyen *et al*. 2015) Li *et al*. 2024, 12^3^	comb. nov. [basonym: *Mesorhizobium soli* Nguyen *et al*. 2015]	NHI-8 (=JCM 19897=LMG 29539)^33^	7	[4]
*Pseudomonas auratipiscis* Duman *et al*. 2024, 11^3^	sp. nov.	119P (=DSM 117162=LMG 33381)	50	[45]
*Pseudomonas carassii* Duman *et al*. 2024, 11^3^	sp. nov.	137P (=DSM 117060=LMG 33378)	50	[45]
*Pseudomonas ulcerans* Duman *et al*. 2024, 11^3^	sp. nov.	147P (=DSM 117163=LMG 33377)	50	[45]
*Reichenbachiella agarivorans* Muhammad *et al*. 2023, 13^3,34^	sp. nov.	BKB1-1 (=JCM 35840=KCTC 82964)^35^	27	[46]
*Reichenbachiella carrageenanivorans* Muhammad *et al*. 2023, 13^3,34^	sp. nov.	WSW4-B4 (=JCM 35841=KCTC 82706)	27	[46]
*Reichenbachiella ulvae* Muhammad *et al*. 2023, 13^3^	sp. nov.	ABR2-5 (=JCM 35839=KCTC 82990)	27	[46]
*Saccharothrix yanglingensis* Yan *et al*. 2012, 144	sp. nov.	Hhs.015 (=CGMCC 4.5627=KCTC 19722)	55	[47]
*Scandinavium lactucae* Park *et al*. 2024, 8^3^	sp. nov.	V105_6 (=DSM 117134=LMG 33389)	21	[48]
*Secundilactobacillus muriivasis* Wang *et al*. 2024, 7^3^	sp. nov.	46-6B (=CCTCC AB 2023259=JCM 36612=LMG 33411)	34	[28]
*Simplicispira sedimenti* Chen *et al*. 2024, 8^3^	sp. nov.	W1-6 (=CGMCC 1.16274=NBRC 115624)	1	[49]
*Sphaerotilus uruguayifluvii* Machin *et al*. 2024, 8^3,36^	sp. nov.	C29 (=CCM 9043=DSM 113250)	13	[50]
*Stenotrophomonas muris* Afrizal *et al*. 2022, e23^3^	sp. nov.	pT2-440Y (=DSM 28631=JCM 35242)^37^	26	[15]
*Streptococcus nakanonensis* corrig. Wajima *et al*. 2024, 9^3,38^	sp. nov.	MTG105 (=CCUG 76894=JCM 35953)	56	[51]
*Streptococcus parapneumoniae* Katayama *et al*. 2024, 6^3,39^	sp. nov.	SP4011 (=JCM 36068=KCTC 21228)	39	[52]
*Streptococcus parasalivarius* Wang *et al*. 2024, 8^3^	sp. nov.	778-2 (=CCTCC AB 2023257=JCM 36614=LMG 33413)	34	[28]
*Streptomyces argyrophylli* corrig. Mo *et al*. 2023, 7^3,40^	sp. nov.	Jing01 (=JCM 35923=MCCC 1K05707)	59	[53]
*Streptomyces poriticola* Kanchanasin *et al*. 2024, 9^3,41^	sp. nov.	C6-003 (=NBRC 116425=TBRC 17807)	53	[35]
*Streptomyces rugosispiralis* Weeraphan *et al*. 2023, 6^3^	sp. nov.	RCU-064 (=NBRC 115861=TBRC 16203)	52	[54]
*Tamlana nanhaiensis* Liu *et al*. 2015, 1195	sp. nov.	FHC16 (=LMG 27420=MCCC 1A06648)^42^	19	[55]
*Teichococcus aerofrigidensis* (Hyeon and Jeon 2017) Liu and Xin 2024, 9^3^	comb. nov. [basonym: *Roseomonas aerofrigidensis* Hyeon and Jeon 2017]	HC1 (=JCM 31878=KACC 19097)	32	[22]
*Teichococcus aerophilus* (Kim *et al*. 2013) Liu and Xin 2024, 10^3^	comb. nov. [basonym: *Roseomonas aerophila* Kim *et al*. 2013]	7515 T-07 (=KACC 16529=NBRC 108923)	32	[22]
*Teichococcus aestuarii* (Venkata Ramana *et al*. 2010) Liu and Xin 2024, 10^3^	comb. nov. [basonym: *Roseomonas aestuarii* Venkata Ramana *et al*. 2010]	JC17 (=KCTC 22692=NBRC 105654)	32	[22]
*Teichococcus cervicalis* (Rihs *et al*. 1998) Liu and Xin 2024, 10^3^	comb. nov. [basonym: *Roseomonas cervicalis* Rihs *et al*. 1998]	E7107 (=ATCC 49957=CIP 104027)	32	[22]
*Teichococcus coralli* (Li *et al*. 2021) Liu and Xin 2024, 10^3^	comb. nov. [basonym: *Roseomonas coralli* Li *et al*. 2021]	M0104 (=KCTC 62359=MCCC 1K03632)	32	[22]
*Teichococcus deserti* (Subhash and Lee 2018) Liu and Xin 2024, 10^3^	comb. nov. [basonym: *Roseomonas deserti* Subhash and Lee 2018]	M3 (=JCM 31275=KEMB 2255̅–459)	32	[22]
*Teichococcus globiformis* (Fang *et al*. 2018) Liu and Xin 2024, 10^3^	comb. nov. [basonym: *Roseomonas globiformis* Fang *et al*. 2018]	CPCC 100847 (=DSM 102171=KCTC 52094)	32	[22]
*Teichococcus hibiscisoli* (Yan *et al*. 2017) Liu and Xin 2024, 10^3^	comb. nov. [basonym: *Roseomonas hibiscisoli* Yan *et al*. 2017]	THG-N2.22 (=CCTCC AB 2016176=KACC 18935)	32	[22]
*Teichococcus musae* (Nutaratat *et al*. 2017) Liu and Xin 2024, 10^3^	comb. nov. [basonym: *Roseomonas musae* Nutaratat *et al*. 2017]	PN1 (=NBRC 107870=TBRC 4638)	32	[22]
*Teichococcus oryzae* (Ramaprasad *et al*. 2015) Liu and Xin 2024, 11^3^	comb. nov. [basonym: *Roseomonas oryzae* Ramaprasad *et al*. 2015]	JC288 (=KCTC 42542=LMG 28711)	32	[22]
*Teichococcus rhizosphaerae* (Chen *et al*. 2014) Liu and Xin 2024, 11^3^	comb. nov. [basonym: *Roseomonas rhizosphaerae* Chen *et al*. 2014]	YW11 (=CCTCC AB 2013041=KACC 17225)	32	[22]
*Teichococcus ruber* (Subhash *et al*. 2016) Liu and Xin 2024, 11^3^	comb. nov. [basonym: *Roseomonas rubra* Subhash *et al*. 2016]	S5 (=JCM 31177=KEMB 563–468)	32	[22]
*Teichococcus suffuscus* (Subhash and Lee 2017) Liu and Xin 2024, 11^3^	comb. nov. [basonym: *Roseomonas suffusca* Subhash and Lee 2017]	S1 (=JCM 31176=KEMB 563–465	32	[22]
*Teichococcus vastitatis* (Zhao *et al*. 2020) Liu and Xin 2024, 11^3^	comb. nov. [basonym: *Roseomonas vastitatis* Zhao *et al*. 2020]	J1A743 (=CPCC 101021=KCTC 62043)	32	[22]
*Teichococcus wenyumeiae* (Tian *et al*. 2019) Liu and Xin 2024, 11^3^	comb. nov. [basonym: *Roseomonas wenyumeiae* Tian *et al*. 2019]	Z23 (=CGMCC 1.16540=DSM 106207)	32	[22]
*Terribium* Li *et al*. 2024, 10^3^	gen. nov.	*Terribium terrae*	7	[4]
*Terribium terrae* (Jung *et al*. 2021) Li *et al*. 2024, 10^3^	comb. nov. [basonym: *Mesorhizobium terrae* Jung *et al*. 2021]	NIBRBAC000500504 (=JCM 33432=KCTC 72278)	7	[4]
*Terrisporobacter muris* Afrizal *et al*. 2022, e23^3^	sp. nov.	CCK3R4-PYG-107 (=DSM 29186=JCM 35245)	26	[15]
*Tessaracoccus lacteus* Tan *et al*. 2024, 6^3^	sp. nov.	T21 (=CCTCC AB 2023031=KCTC 49936)	20	[56]
*Thermanaerothrix* Grégoire *et al*. 2011, 407^43^	gen. nov.	*Thermanaerothrix daxensis*	5	[57]
*Thermanaerothrix daxensis* Grégoire *et al*. 2011, 407^44^	sp. nov.	GNS-1 (=DSM 23592=JCM 16980)	5	[57]
*Thermanaerothrix solaris* Zayulina *et al*. 2024, 10^3^	sp. nov.	4228-RoL (=BIM B-2058=UQM 41594)^45^	6	[58]
*Thioclava litoralis* Chen *et al*. 2024, 9^3^	sp. nov.	FTW29 (=KCTC 82841=MCCC 1K08523)^46^	24	[59]
*Trichlorobacter ammonificans* Sorokin *et al*. 2024, 1^3^	sp. nov.	G1 (=DSM 105480=UNIQEM U1005)	41	[60]
*Undibacterium cyanobacteriorum* Van Le *et al*. 2024, 7^3^	sp. nov.	20NA775 (=LMG 33136=KCTC 8005)	17	[61]
*Yunchengibacter* Guo *et al*. 2025, 8^3^	gen. nov.	*Yunchengibacter salinarum*	49	[62]
*Yunchengibacter salinarum* Guo *et al*. 2025, 8^3^	sp. nov.	YC-2023-2=GDMCC 1.4502=KCTC 8546)	49	[62]

For references to Validation Lists 1–71, see *Int J Syst Bacteriol* **49** (1999) 1325. Lists 72–197 were published in *Int J Syst Evol Microbiol* **50** (2000) 3, 423, 949, 1415, 1699, 1953; and **51** (2001) 1, 263, 793, 1229, 1619, 1945; and **52** (2002) 3, 685, 1075, 1437, 1915; and **53** (2003) 1, 373, 627, 935, 1219, 1701; and **54** (2004) 1, 307, 631, 1005, 1425, 1909; and **55** (2005) 1, 547, 983, 1395, 1743, 2235; and **56** (2006) 1, 499, 925, 1459, 2025, 2507; and **57** (2007) 1, 433, 893, 1371, 1933, 2449; and **58** (2008) 1, 529, 1057, 1511, 1993, 2471; and **59** (2009) 1, 451, 923, 1555, 2129, 2647; and **60** (2010) 1, 469, 1009, 1477, 1985, 2509; **61** (2011) 1, 475,1011, 1499, 2025, 2563; and **62** (2012) 1, 473, 1017, 1443, 2045, 2549; and **63** (2013) 1, 797, 1577, 2365, 3131, 3931; and **64** (2014) 1, 693, 1455, 2184, 3603; and **65** (2015), 1, 741, 1112, 2017, 2777, 3767; and **66** (2016) 1, 1603, 1913, 2463, 3761, 4299; and **67** (2017) 1, 529, 1095, 2075, 3140, 4291; and **68** (2018), 1, 693, 1411, 2130, 2707, 3379; and **69** (2019), 5, 597, 1247, 1844, 2627, 3313; and **70** (2020) 1, 1443, 2960, 4043, 4844, 5596. Lists 197–220 were published in *Int J Syst Evol Microbiol* **71** (2021) 004600, 004688, 004773, 004846, 004943, 005096; and **72** (2022) 005167, 005260, 005331, 005422, 005517, 005592; and **73** (2023) 005709, 005812, 005845, 005931, 005997, 006080; and **74** (2024) 006173, 006229, 006275, 006398, 006452, 006501.

1 Abbreviations of culture collections cited in this list can be found at https://lpsn.dsmz.de/text/collections.

2 Priority numbers are assigned according to the date the documentation and request for validation are received.

3 The journal in which the name was effectively published does not have continuous pages.

4 The ACCC number 14561, correctly given in the description of the basonym, was misprinted 15461.

5 The effective publication states that the type strain was also deposited as ACCC 14549, but no documentation was supplied.

6 The etymology should state N.L. neut. adj. instead of N.L. neut. Adj.

7 The list editors have corrected the epithet *lansinium* to *lansingense* (lan.sing.en’se. N.L. neut. adj. *lansingense*, pertaining to Lansing, MI, USA).

8 The authors gave TSD 326 instead of ATCC TSD-326.

9 The basonym was assigned to Risk Group 2 [German TRBA 466, ‘Einstufung von Prokaryoten (Bacteria und Archaea) in Risikogruppen’].

10 The effective publication states that the type strain was also deposited as ATCC 33650 and NBRC 105704, but no documentation was supplied.

11 The list editors have corrected the epithet to *subterraneus* (sub.ter.ra’ne.us. L. masc. adj. *subterraneus*, underground, subterranean).

12 The effective publication states that the type strain was also deposited as CCUG 77273, but no documentation was supplied.

13 The effective publication states that the type strain was also deposited as CCUG 68796, but no documentation was supplied.

14 The preferred syllabification is a.xi.nel’lae.

15 The preferred syllabification and etymology are wel.lin’gi.i. N.L. gen. n. *wellingii*, …

16 The ACCC document gives strain number 6GGN-30 instead of 6GN-30.

17 The preferred syllabification and etymology are wan.ju.en’sis. N.L. fem. adj. *wanjuensis*, …

18 The preferred etymology is N.L. neut. n. *manganum*, manganese; …

19 The effective publication states that the type strain was also deposited as SDUM 032109, but no documentation was supplied.

20 The effective publication states that the type strain was also deposited as SDUM032111, but no documentation was supplied.

21 The list editors have corrected the epithet to *salinarum* (sa.li.na’rum. L. pl. gen. n. *salinarum*, of salt works).

22 The effective publication states that the type strain was also deposited as JCM 3076, IFO 14624, NBRC 14624 and VKM Ac-1507, but no documentation was supplied.

23 The effective publication states that the type strain was also deposited as KEMB 9005-416, but no documentation was supplied.

24 The effective publication states that the type strain was also deposited as CIP 110074 and DSM 25622, but no documentation was supplied.

25 The effective publication states that the type strain was also deposited as CIP 110074 and CCM 7468, but no documentation was supplied.

26 The list editors have corrected the epithet *landrumus* to *landrumensis* (lan.drum.en’sis. N.L. masc. adj. *landrumensis*, pertaining to Landrum, SC, USA).

27 The authors gave TSD-327 instead of ATCC TSD-327. The effective publication states that the type strain was also deposited as NRRL B-65669, but no documentation was supplied.

28 The list editors have corrected the epithet *poenicansa* to *punicea* (pu.ni’ce.a. L. fem. adj. *punicea*, red).

29 The authors gave TSD-332 instead of ATCC TSD-332.

30 The effective publication states that the type strain was also deposited as USDA 4964, but no documentation was supplied.

31 The preferred syllabification and etymology are pi.tu.i'ta. L. fem. n. *pituita*, mucus, referring to the colony texture of the type strain).

32 The syllabification must read com.pos’ti. instead of com.posAAti.

33 The effective publication states that the type strain was also deposited as KACC 17916.

34 The etymology should state N.L. part. adj. instead of N.L. part. Adj.

35 The KTCC document states strain number BKBl-1 instead of BKB1-1.

36 The preferred etymology is L. masc. n. *fluvius*, river; N.L. gen. n. *uruguayifluvii*, of the Uruguay River.

37 The culture collection documents state strain designation pTZ-440Y instead of pT2-440Y given in the paper.

38 The list editors have corrected the name to *Streptococcus nakanonensis* (na.ka.no.nen’sis. N.L. masc. adj. *nakanonensis*, pertaining to Nakano).

39 The authors did not provide the etymology. We propose the following syllabification and etymology: pa.ra.pneu.mo’ni.ae. Gr. prep. *para*, alike, alongside of; N.L. gen. n. *pneumoniae* (‘of pneumonia’), specific epithet of a *Streptococcus* species; N.L. gen. n. *parapneumoniae*, alike (*Streptococcus*) *pneumoniae*.

40 The list editors have corrected the epithet to *argyrophylli* (ar.gy.ro.phyl’li. Gr. masc. n. *argyros*, silver; Gr. neut. n. *phyllon*, leaf; N.L. gen. n. *argyrophylli*, of a silver leaf, referring to *Cathaya argyrophylla*, from which the type strain was isolated.

41 In the protologue heading, the specific epithet must not be capitalized.

42 The effective publication states that the type strain was also deposited as CGMCC 1.12469, but no documentation was supplied.

43 The preferred syllabification is Therm.an.ae’ro.thrix.

44 The protologue must state *Thermanaerothrix daxensis* instead of *T. daxensis*.

45 The effective publication states that the type strain was also deposited as VKM M-3776, but no documentation was supplied.

46 The authors gave KCTC 82,841 instead of KCTC 82841.

## Abbreviations

ICNP: International Code of Nomenclature of Prokaryotes
*IJSEM*: *International Journal of Systematic and Evolutionary Microbiology*
